# *Thymus serpyllum* Exhibits Anti-Diabetic Potential in Streptozotocin-Induced Diabetes Mellitus Type 2 Mice: A Combined Biochemical and In Vivo Study

**DOI:** 10.3390/nu14173561

**Published:** 2022-08-29

**Authors:** Jahanzaib Azhar, Peter John, Attya Bhatti

**Affiliations:** Department of Healthcare Biotechnology, Atta-Ur-Rahman School of Applied Biosciences (ASAB), National University of Sciences and Technology (NUST), Islamabad 44000, Pakistan

**Keywords:** *Thymus serpyllum*, type 2 diabetes, glucose and insulin tolerance, *AMPK*, *IRS1*

## Abstract

Type 2 diabetes mellitus (T2DM) is a complex metabolic disorder that is characterized by hyperglycemia, insulin resistance, and lack of insulin production. It has been previously reported that *Thymus serpyllum* has therapeutic potential against many diseases. To investigate the antidiabetic action of *Thymus serpyllum*, this study aimed to analyze its restorative impact in diabetic mice, in which it was administered in diet. Diabetes was induced in BALB/c mice fed with a high-fat diet and two intraperitoneal injections of streptozotocin. With the onset of diabetes, the mice were administered daily with aqueous extract of *Thymus serpyllum* (500 mg/kg/d and 800 mg/kg/d) for 4 weeks. Body weight and fasting blood glucose levels were measured after every 1 week of the treatment. Subsequently, intraperitoneal glucose tolerance and insulin tolerance tests were conducted. In addition, liver tissue was isolated for assessment in terms of levels of gene expression of the *AMPK*, *IRS1*, and *GLUT2* gene. Treatment with the aqueous extract of *Thymus serpyllum* was found to be significantly effective in controlling hyperglycemia and improving glucose and insulin tolerance. Predictable with these impacts, the extract of *Thymus serpyllum* upregulated the *AMPK* expression at the mRNA level, as well as upregulating the expression of *IRS1* and *GLUT2* gene. Histopathological examination of the liver, kidney, and pancreas also revealed the restorative impact in terms of cellular morphology. The results hence demonstrated that oral administration of aqueous extract of *Thymus serpyllum* can potentially attenuate hyperglycemia in the liver muscle of streptozotocin (STZ)-induced diabetic mice via *AMPK* and *IRS1* upregulation.

## 1. Introduction

Type 2 diabetes mellitus is a complex metabolic disease which is characterized by high blood plasma glucose levels due to insufficient production of insulin, defective signaling, and inefficient insulin action in the body [1,2]. It is estimated that globally about 1 in 11 adults suffer from diabetes and 90% of cases are of type 2 diabetes (T2DM) [3]. The complex pathogenesis of type 2 diabetes is not very clear but it is widely accepted that insulin resistance and impaired insulin secretion are the major hallmarks of type 2 diabetes. In insulin resistance, body tissues are unable to sense insulin and insulin becomes unable to take glucose inside the cells for glucose homeostasis in the body [4]. Glucose homeostasis in the body depends on many factors including the regulation of various pathways such as insulin signaling, adipocytokine, PI3K/AKT, and the *AMPK* pathway. AMP-activated protein kinase (*AMPK*) has been shown to regulate and monitor energy processes at cell and tissue level [5]. *AMPK* regulates the hepatic glucose production and hepatic lipid metabolism in the body by metabolizing glucose through one of the processes in which *AMPK* suppressed the levels of expression of two enzymes including phosphoenolpyruvate carboxykinase as well as glucose-6-phosphate [6,7]. Inhibition of these two enzymes in turn inhibits gluconeogenesis [8]. Insulin receptor substrate 1 (*IRS1*) is another major protein involved in maintaining glucose levels in the body by regulating insulin signaling in the body. Phosphorylation of *AMPK* activates the insulin receptor, providing a direct link between *AMPK* and insulin signaling as this pathway promotes energy conservation and survival of muscle exposed to severe glucose deprivation [9]. It has been also reported that in mice and patients with type 2 diabetes, there are low levels of *IRS1* and *AMPK*, both at RNA and protein level [10,11]. Moreover, *GLUT2* expression is required for the physiological control of glucose-sensitive genes, and its inactivation in liver leads to impaired glucose-stimulated insulin secretion [12].

T2DM can be managed with drugs, insulin therapy, and improving lifestyle. There are different classes of drugs that have been approved for treatment of type 2 diabetes including biguanides, thiazolidinedione, DPP4 inhibitors, sulfonylureas, and alpha glucosidase inhibitors. Unfortunately, these drugs create the serious problem of resistance and side effects such as abdominal discomfort, diarrhea, anorexia, balanoposthitis, and urogenital tract infections [13,14]. Metformin is one of the most popular oral glucose-lowering medications, widely considered to be the optimal initial therapy for patients with type 2 diabetes mellitus [15]. Physiologically, metformin has been shown to reduce hepatic glucose production by acting on the liver via *AMPK* activation [16] but now literature has been moved to a much more complex picture reflecting its multiple modes of action. Particularly, in addition to inhibiting hepatic glucose production, metformin has also been shown to act on skeletal muscle to increase insulin-stimulated glucose uptake [17,18]. Furthermore, the therapeutic efficacy of metformin has been linked to alterations in gut microbiome composition, intestinal glucose uptake, and glucagon-like peptide-1 (GLP-1) hormone secretion [19]. Metformin also creates adverse effects of a gastrointestinal nature, such as diarrhea, nausea, and abdominal discomfort. These effects are usually mild, transient, and dose-related, but can occur in up to 50% of people taking the drug [20]. Therefore, by virtue of the side effects of medications and drug resistance problems in T2DM, new treatment options are under strict investigation.

*Thymus serpyllum*, locally known as ‘tomoru’, is an indigenous plant of the Himalayan range. It belongs to family Laminaceae and genus thymus that have significant ethnobotanical importance. *Serpylli herba* is an official drug in the European Pharmacopeia composed of the dried flower aerial parts of *Thymus serpyllum*. Traditionally, dried leaves and flowers of the herb were used to prepare tea and infusions against cold, bronchitis, fever, and cough, thus indicating its safety in humans [21]. The medicinal efficacy of *Thymus serpyllum* extract has mainly been focused on its essential oil obtained by steam distillation but the crude extract has also been evident to treat respiratory tract infections and other complications [22]. Additionally, *Thymus serpyllum* extract has been explored for its anti-tumor, antioxidant, anti-inflammatory, anti-microbial, and cytotoxic properties against multi-drug resistant bacteria, cancer, and other autoimmune disorders, in which the presence of phenolic derivatives seems to play a key role [23,24,25,26]. Despite being famous for its efficacy against inflammatory conditions, the potential benefits of *Thymus serpyllum* against type 2 diabetes are rarely investigated. Therefore, the present study was focused on evaluating the therapeutic efficacy of *Thymus serpyllum* to reduce hyperglycemia in streptozotocin-induced diabetic mice models. To elucidate this, aqueous extract was prepared and administered orally to the model. Treatment proves to be effective against the disease and improves the glucose and insulin tolerance in vivo.

## 2. Material and Methods

### 2.1. Plant Collection and Extract Preparation

**Figure 1:**
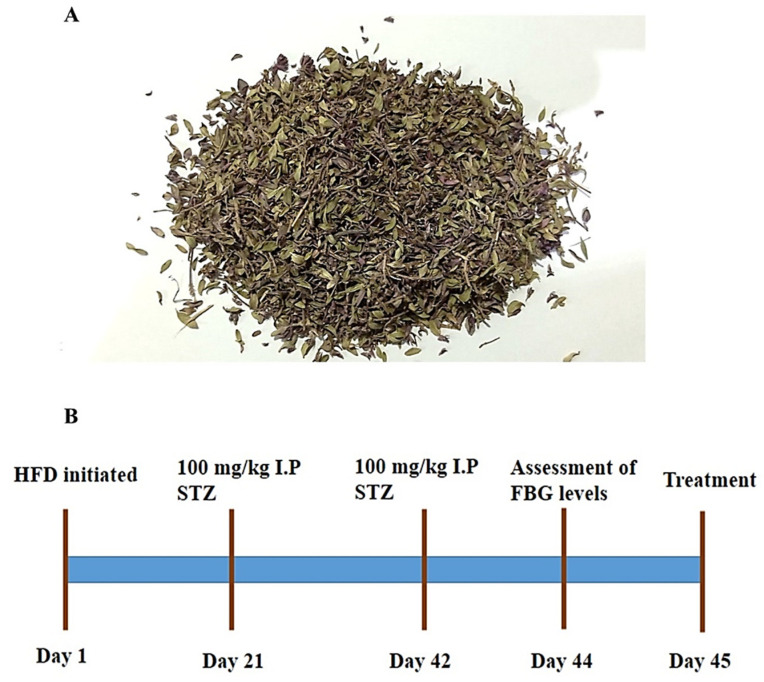
(**A**) Image of the *Thymus serpyllum*, harvested for the present study. (**B**) Timeline depicting the diabetic mice model construction. HFD: High-fat diet; STZ: Streptozotocin; FBG: Fasting blood glucose.

*Thymus serpyllum* was collected from the area Nagar valley, Rakaposhi base camp, Gilgit Baltistan, Pakistan (Figure 1A). Plant material was ground to make its fine powder and dissolved by 1:10 in distilled water. Then, the extract was left for two weeks in a dark area for maceration and was shaken routinely 2–3 times. After 2 weeks of maceration, the extract was filtered with the help of Whatman filter paper and purified to remove chlorophyll content using organic solvent extraction as described by [27]. The filtrate was shifted to a rotary evaporator for solvent evaporation to have final solid form of the extract. Afterwards, extract was stored at 4 °C until further use in the treatment of mice models.

### 2.2. Phytochemicals Screening

Phytochemical screening was as previously done by Jannat et al., 2019 [24] to evaluate the constituents present in the *Thymus serpyllum* aqueous extract. The strength of presence was measured by comparing with extracts of *Trachyspermum ammi*, which is known to be rich in phytochemicals [28].

### 2.3. Antioxidant and Alpha Amylase Inhibition Assay

Antioxidant activity of chlorophyll removed extract was evaluated through di-phenyl-2-picryl hydrazyl hydrate (DPPH) assay using protocol followed by Sanganna et al. [29] with few modifications. Briefly, the extract was diluted in different concentrations and 20–80 μg concentrations of extract were adopted. The same concentrations were adopted for the ascorbic acid which acts as a control. The DPPH solution was made in methanol in 5:100. The volume of 1 mL for each concentration of extract and control was prepared in microfuge tube and 0.5 mL DPPH solution was added. The reaction mixture was incubated for 1 h at room temperature in dark. After incubation, the optical density (OD) was taken at 517 nm by blanking with the solvent that was used in the extract and control preparation.

Alpha amylase inhibition was done following the protocol of McCue and Shetty [30] with slight modifications. In brief, 20–80 μg concentrations of extract and control (acarbose) were prepared. The alpha amylase solution was prepared as 0.5 mg/mL in 0.02 M sodium phosphate buffer. The 250 μL enzyme solution was added in 250 μL volume of each concentration of control and extract. After the incubation of 10 min at room temperature, 250 μL volume of the 1% starch solution made in 0.02 M sodium phosphate buffer was added and further incubated for 10 min. The reaction was stopped by adding 500 μL of DNS reagent. The reaction tubes were kept in the boiling water bath for 5 min and then cooled at room temperature. A measure of 5 mL of distilled water was added in each concentration of extract and control to dilute the solution. The optical density was measured at 520 nm. The percentage % inhibition for both experiments was calculated by the Formula (1).

(1) $$\%\;\mathrm{Inhibition}=\frac{\mathrm{Abs}\;\mathrm{control}-\mathrm{Abs}\;\mathrm{sample}}{\mathrm{Abs}\;\mathrm{control}}\times 100\;$$

### 2.4. In Vivo Assessment of Antidiabetic Potential

#### 2.4.1. Animal Procurement and Model Establishment

Male BALB/c mice of 3–4-week age were used and kept in a pathogen free environment at the animal house ASAB, National University of Sciences and Technology. All the experiments and protocols followed were approved by Institutional Review Board at ASAB (IRB-ASAB). The animals were provided with basic mice feed and tap water. All the experiments were carried out according to the National Institutes of Health’s Guide for the Care and Use of Laboratory Animals.

The mice model of T2DM was established by combination of high fat diet (HFD) and two doses of streptozotocin (STZ) injection as previously reported by Noor et al. [31]. The timeline depicting the mice model construction period is explained in Figure 1B. A total of 50 mice, after being weaned when they were 3 weeks of age, were taken. The group of mice that served as normal control (*n* = 10) received basic feed ((crude protein 30%, crude fat 9%, and crude fiber 4%) while the others (*n* = 40) received a high fat diet (basic mice feed 59%, sugar 20%, animal fat 18%, and egg yolk 3%) daily. Streptozotocin dissolved in 0.1 M citrate buffer (PH 4.5) was given intraperitoneally at a dose of 100 mg/kg on the 6th and 9th week age of mice. Before the injection, the mice were kept fasting for 12 h, then they were injected with 100 mg/kg streptozotocin while those in the normal group were injected with only citrate buffer. Following 48 h after the 2nd injection, the glucose levels of mice were measured by Glucometer (On Call-Acon Labs Inc. San Diego, CA 92121, United States). Afterwards, mice were classified into 5 groups for treatment and further experiments, as follows.

Group 1: Normal control mice (*n* = 10).
Group 2: Diabetic or untreated mice group (*n* = 10).
Group 3: Mice group treated with standard drug metformin at 100 mg/kg dose (*n* = 10).
Group 4: Mice group treated with 500 mg/kg dose of *Thymus serpyllum* extract (*n* = 10).
Group 5: Mice group treated with 800 mg/kg dose of *Thymus serpyllum* extract (*n* = 10).

#### 2.4.2. Administration of *Thymus serpyllum* for Treatment

Measures of 500 mg/kg and 800 mg/kg of aqueous extract were administered orally through feed, respectively, for 4 weeks. The concentration of dose was selected by considering LD50 value calculated by previous experiments (data not shown). From animal dose, the human equivalent dose (HED) can be calculated using Formula (2) as previously done by Shin et al. [32]. Metformin was used as the standard drug because it is usually the first medication given for hyperglycemic condition in type 2 diabetes. Moreover, metformin has a pleiotropic activity and favorable effects in diabetes-associated complications. Metformin was given to the mice at 100 mg/kg dose. During the treatment, body weight and fasting blood glucose levels were measured on days 7, 14, 21, and 28.

(2) $$\mathrm{HED}\;\left(\frac{\mathrm{mg}}{\mathrm{kg}}\right)=\;\mathrm{Animal}\;\mathrm{dose}\frac{\mathrm{mg}}{\mathrm{kg}}\times {\left[\frac{\mathrm{Animal}\;\mathrm{weight}\;\left(\mathrm{kg}\right)}{\mathrm{Human}\;\mathrm{weight}\;\left(\mathrm{kg}\right)}\right]}^{0.33}$$

#### 2.4.3. Glucose and Insulin Tolerance Test

On day 29, the mice were kept fasting overnight and the next morning they were given with glucose load of 2 g/kg to evaluate their ability to tolerate glucose in the body. For this purpose, the glucose level of mice models was measured before injection and values were recorded for 0 min. After this, each mouse was given an intraperitoneal injection of glucose at a dose of 2 g/kg and their glucose levels were measured at 30, 60, 90, and 120 min. The area under the curve for glucose levels in different intervals of time was also measured.

Likewise, on day 31, the mice models were injected with insulin to assess the insulin sensitivity in their body. For this purpose, mice were kept fasting overnight and the next morning, they were injected with 0.5 U/kg insulin intraperitoneally. The blood glucose measurements were taken at 0 min before injection and then measured at 30, 60, 90, and 120 min. The area under the curve for glucose level in different intervals of time was also measured.

#### 2.4.4. Expression Analysis of *AMPK*, *IRS1* and *GLUT2* gene by Quantitative Real-Time Polymerase Chain Reaction (RT-PCR)

To evaluate the effect of aqueous extract of *Thymus serpyllum* on the expression of *AMPK*, *IRS1* and *GLUT2* gene, total RNA was isolated from liver tissue using TRIzol reagent and cDNA was synthesized using first strand cDNA synthesis kit (ThermoFisher, Waltham, MA, USA). Quantitative detection of these specific genes was carried out in real time PCR (Applied Biosystem, Bedford, MA, USA). The primer pairs that were used are listed in (Table S1, Supplementary Materials). All samples for q-PCR were assayed in triplicate, and data were normalized to the relative levels of GAPDH as a housekeeping gene in the same experiment.

#### 2.4.5. Histopathological Examination

Specimens of liver, pancreas, and kidney were collected and fixed in 10% formalin solution. In addition, 70%, 80%, and 100% concentrations of ethanol were prepared into which the liver, kidney, and pancreas were immersed for 2 h each. Afterwards, the samples were cleared in xylene and embedded in paraffin. After drying, samples were sectioned through microtome and then the section ribbon was fixed on a slide, using a hot and cold water bath cycle, for staining. Using hematoxylin and eosin, slides were stained after rehydration and then sealed with DPX mountant and coverslip, after which they were observed with light microscope at 4×, 10× and 40× to observe changes amongst the study groups.

### 2.5. Statistical Analysis

Statistical analysis was performed using the GraphPad Prism. The results are expressed as mean ± standard deviation. The comparison between different groups was done by one-way analysis of variance and two-way analysis of variance (ANOVA). The Bonferroni test was used for testing the inter-grouping homogeneity. Statistical significance was set *p* < 0.05.

## 3. Results

### 3.1. Phytochemicals Screening, Antioxidant and Alpha Amylase Inhibition Activity

**Figure 2:**
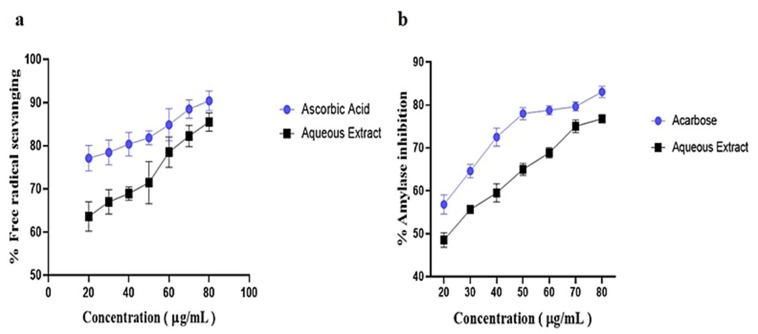
Antioxidant and alpha amylase inhibition activity. (**a**) The free radical scavenging activity of aqueous extract increased with the increasing concentration of the extract showing its inhibitory potential for free radicals. (**b**) The potential of aqueous extract to inhibit alpha amylase increased with the increasing concentration of the extract showing that it can manage postprandial hyperglycemia.

Aqueous extract of *Thymus serpyllum* (TS-AQ) was shown to be rich in flavonoids, phenols, coumarins, alkaloids, and many other classes of phytochemicals including terpenoids, anthocyanins, and glycosides (Table 1). When it was evaluated for its antioxidant activity, the extract showed a progressive pattern of its ability to scavenge free radicals concerning increased concentration. There was a direct linear relationship between free radical scavenging activity and increased aqueous extract and control concentrations (Figure 2a). Similarly, when the aqueous extract was evaluated for its ability to inhibit alpha amylase, a continuous trend was observed in the case of both aqueous extract and control (acarbose), which shows the direct linear correlation of % inhibition with increased concentration (Figure 2b). Hence, biochemical analysis revealed that *Thymus serpyllum* aqueous extract can be a potential antioxidant and can be used to manage post-prandial hyperglycemia via inhibition of alpha amylase.

### 3.2. Thyme Extract Reduced Fasting Blood Glucose Levels in BALB/c Mice

**Figure 3:**
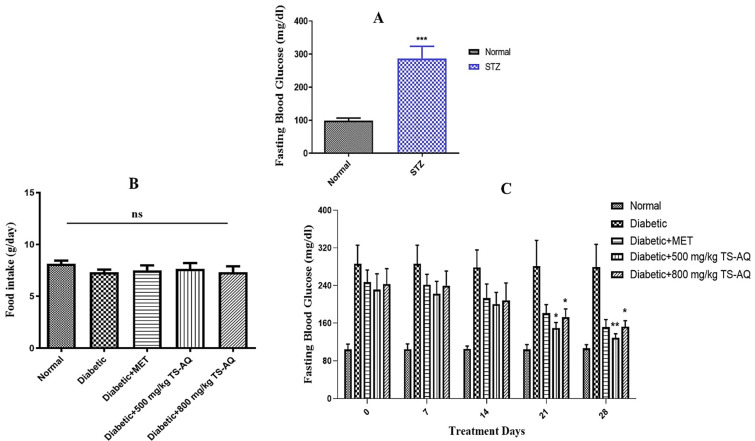
Effect of aqueous extract of thyme on fasting blood glucose levels. (**A**) Blood glucose level of STZ group vs. Normal. The animals injected with streptozotocin (STZ) showed higher levels of blood glucose as compared to normal confirming the induction of diabetes mellitus. (**B**) Average daily food intake. (**C**) Fasting blood glucose (FBG) levels among different groups after every 1 week of the treatment. All the treated groups showed significant reduction in FBG levels after the 3rd and 4th week of the treatment. ns = not significant. * Depict statistical significance. * = *p* < 0.05. ** = *p* < 0.01. *** = *p* < 0.001. (*n* = 10 for each group).

Before the treatment, the animals were tested for blood glucose levels for confirmation of STZ-induced hyperglycemia. The STZ group showed a significantly higher level of fasting blood glucose compared to normal (Figure 3A). Thyme extract was administered daily for 4 weeks, and food intake was found to be similar in all treated groups throughout the experimental period (Figure 3B). During the treatment, the daily administration of thyme extract reduced hyperglycemia among all treated groups and it was evaluated that both doses of aqueous extract 500 mg/kg and 800 mg/kg significantly reduced fasting blood glucose levels in STZ-induced diabetic mice compared to untreated group which showed continuous higher pattern of glucose levels (Figure 3C). Metformin also showed improved glucose homeostasis during the treatment.

### 3.3. Effect of Thyme Extract on Body Weight

**Figure 4:**
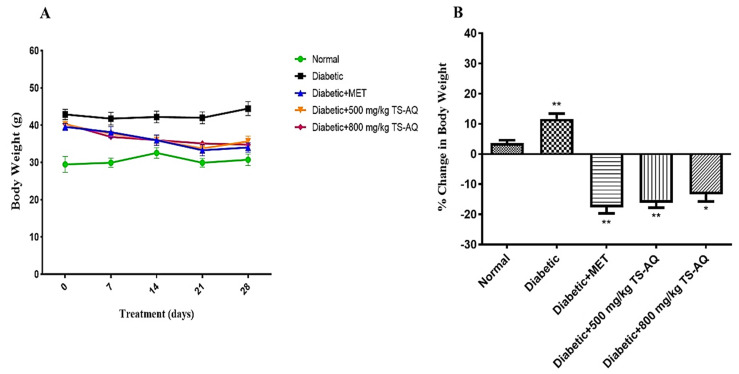
Effect of thyme extract on body weight. (**A**) Measurement of body weight after every 1 week of the treatment. (**B**) The % change in body weight of each group was calculated by the formula: (body weight on day 28 −, body weight on day 0)/body weight on day 0 × 100. * Depict statistical significance. * = *p* < 0.05. ** = *p* < 0.01. (*n* = 10 for each group).

Twenty-eight days of thyme extract supplementation showed gradual reduction in body weight among the treated groups. At the initial time point, no significant difference in body weight was observed. However, after 3rd and 4th week, the body weight of the treated groups was lower than those of their peers in untreated group (Figure 4A). The percentage % change in body weight of all groups was also calculated and expressed in (Figure 4B). Mice treated with metformin showed 17% reduction in body weight. In contrast, mice treated with 500 mg/kg and 800 mg/kg of thyme extract showed 16% and 13% reduction in their body weight, respectively.

### 3.4. Thyme Extract Improved Glucose Tolerance In Vivo

**Figure 5:**
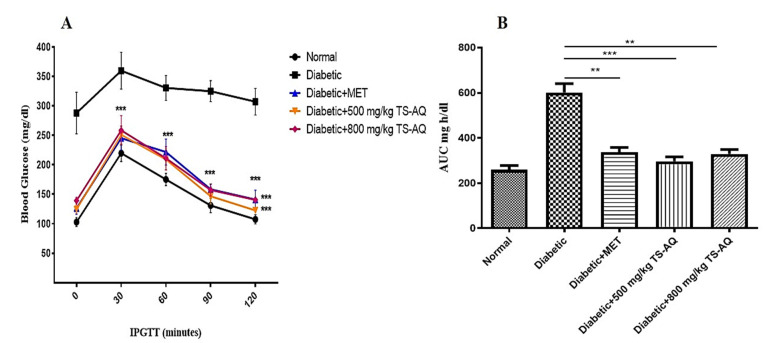
Intraperitoneal glucose tolerance test. (**A**) Blood glucose levels of different groups at 30, 60, 90, and 120 min after intraperitoneal load of glucose (2 g/kg). Before injection, values for the blood glucose levels were considered as 0 min. (**B**) The AUC curve showing the glycemic index for each group. ** = *p* < 0.01. *** = *p* < 0.001. (*n* = 10 for each group).

Impaired tolerance of glucose and insulin is known to be a characteristic of insulin resistance. When the mice were evaluated for the glucose tolerance test, the untreated group showed impaired glucose tolerance when compared to the normal. Both 500 mg/kg and 800 mg/kg doses of extract improved the glucose tolerance when compared with untreated control (*p* =≤ 0.001) (Figure 5A). The glycemic index in each group was expressed and monitored as area under the curve (AUC) (Figure 5B).

### 3.5. Effect of Thyme Extract on Insulin Action

**Figure 6:**
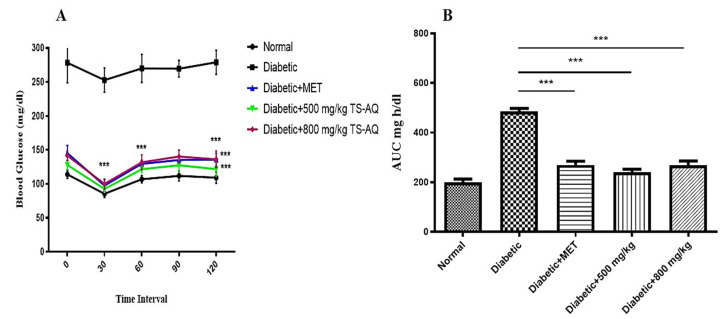
Insulin tolerance test. (**A**) Blood glucose levels were assessed after intraperitoneal injection of insulin (0.5 U/kg) at 30, 60, 90, and 120 min. Before injection, values were recorded for 0 min. (**B**) The AUC curve showing the glycemic index for each group. *** = *p* < 0.001. (*n* = 10 for each group).

To investigate the effect of aqueous extract on insulin action, the insulin tolerance test was conducted. The untreated group was unable to lower their blood glucose level showing the characteristic of insulin resistance, while the metformin and both doses of aqueous extract (500 mg/kg and 800 mg/kg) improved the insulin sensitivity in the body of all treated groups (Figure 6A). The glycemic index was measured and expressed as the area under the curve (AUC) (Figure 6B).

### 3.6. Thyme Extract Improved the AMPK, IRS1 and GLUT2 Expression at RNA Level

**Figure 7:**
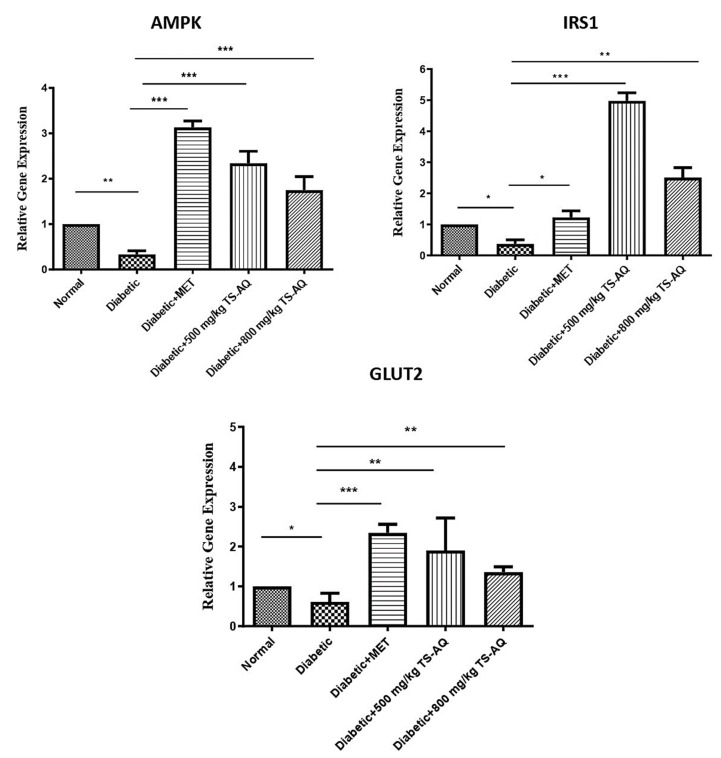
Expression analysis of *AMPK*, *IRS1*, and *GLUT2* genes. The expression of *AMPK*, *IRS1*, and *GLUT2* genes was upregulated after the administration of aqueous extract. The metformin-treated groups showed similar effect in *AMPK* and *GLUT2* gene expression but little improvement in *IRS1* signaling when compared to untreated control. Diabetic or untreated control showed downregulation of genes when compared to normal mice group. Data were obtained from three independent observations and presented as mean ± SD. * = *p* < 0.05. ** = *p* < 0.01. *** = *p* < 0.001.

To investigate the mechanism of action, the effect of aqueous extract on gene expression was evaluated through RT-PCR methodology. From Figure 7, it is evident that gene expression of *AMPK*, *IRS1*, and *GLUT2* gene was downregulated in the untreated group as compared to the normal control. The metformin-treated group showed significant upregulation of the *AMPK* and *GLUT2* genes but little improvement in the upregulation of IRS1. In addition, 500 mg/kg and 800 mg/kg aqueous extract of *Thymus serpyllum* showed marked increase in expression of both *AMPK* and *IRS1* genes when compared to the untreated group. The extract was also evident to normalize the levels of *GLUT2* in treated groups. This showed that aqueous extract may enhance the working of these genes which in turn could enhance the insulin signaling in liver tissue to manage glucose homeostasis. Activation of the *AMPK* gene can trigger the *IRS1* regulation directly or indirectly for active insulin signaling response and subsequent glucose utilization by the tissue.

### 3.7. Effect of Extract on Liver, Kidney, and Pancreas

**Figure 8:**
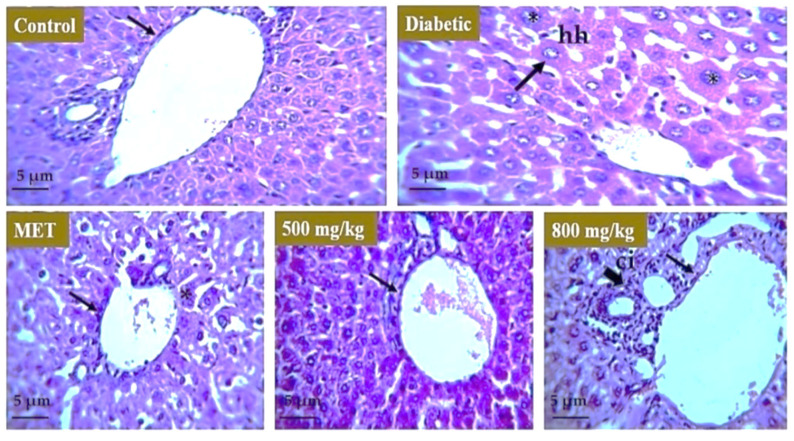
Effect of aqueous extract on the liver tissue. The liver of control mice group revealed intact hepatic portal vein and no hypertrophy in cells. Diabetic mice liver revealed hepatocyte hypertrophy and vein distortion (arrows). Metformin-treated mice revealed improvement in morphology of liver tissue (*). Furthermore, 500 mg/kg TS-AQ dose-treated mice showed restoration of normal structure of liver and size of hepatocyte was restored (*), and 800 mg/kg TS-AQ-treated mice revealed improvement in restoration of normal morphology of liver but there was some cellular infiltration (arrow). **hh** = hepatocyte hypertrophy. **ci** = cellular infiltration.

**Figure 9:**
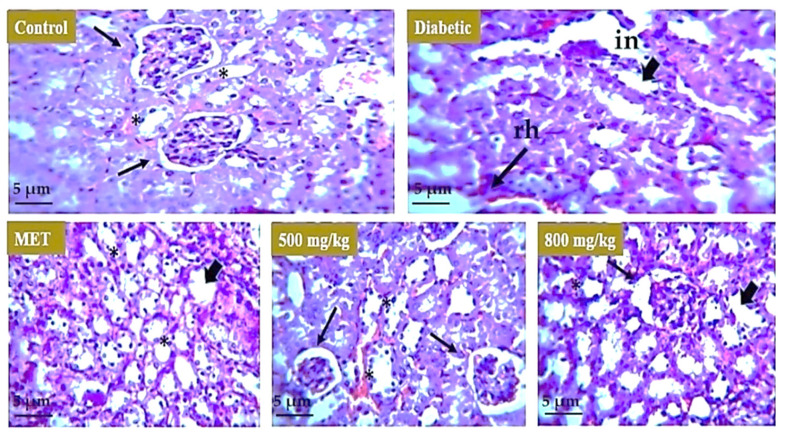
Effect of aqueous extract on the kidney. The kidney of the normal mice revealed intact glomeruli and tubular structure. The kidney of diabetic mice showed interstitial nephritis and renal hemorrhage (arrows). Metformin-treated mice showed little improvement in interstitial nephritis and renal hemorrhage. The 500 mg/kg TS-AQ dose-treated mice revealed significant improvement toward normal glomeruli structure and restoration in normal renal morphology (*). The 800 mg/kg TS-AQ-treated mice also showed little improvement in restoring kidney normal internal structure and tubules. **in** = interstitial nephritis. **rh** = renal hemorrhage.

**Figure 10:**
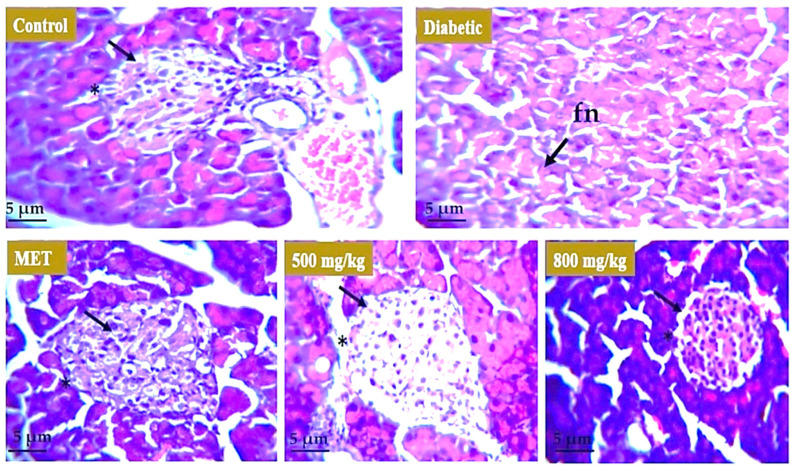
Effect of aqueous extract on the pancreatic tissue. Pancreas of normal mice revealed intact boundary and normal morphology of B cells. The diabetic mice group revealed focal necrosis (arrow) in pancreatic tissue due to the effect of STZ. Mice treated with metformin showed restoration of intact structure of islets of Langerhans. Both 500 mg/kg and 800 mg/kg doses of extract restored the boundary of islets of Langerhans as well as the morphology and number of beta cells (*). **fn** = focal necrosis.

Histopathological analysis was carried out to evaluate the effect of aqueous extract on the liver, kidney, and pancreatic tissue. For the liver (Figure 8), the healthy group represents the normal morphology of liver. The boundary of the hepatic portal vein was intact, and the size of the hepatocytes was normal. In the model or untreated group, the liver morphology showed distortion and there was hypertrophy observed in hepatocytes. In animal groups treated with metformin, 500 mg/kg and 800 mg/kg aqueous extract restored the boundary of hepatic portal vein and size of hepatocytes moved toward normal but there was some cellular infiltration observed in the 800 mg/kg treated group. For the kidney (Figure 9), the healthy group represents the normal morphology of kidney with normal structure and boundary of glomerulus. Renal tubules were also normal in size. The model or untreated group showed distorted morphology of the kidney as boundary of glomeruli was degenerated, showing renal hemorrhage, and interstitial nephritis was observed in tissue. There were symptoms of diabetic nephropathy in the diabetic group. The 500 mg/kg aqueous extract showed improved morphology of the kidney as the boundary of glomeruli was restored and there was also improvement in renal hemorrhage. The effect of metformin and 800 mg/kg extract was not as significant as the tubular structure was improved but the boundary of glomeruli was distorted. For the pancreas (Figure 10), the healthy group showed normal structure of pancreatic tissue and boundary around the islets of Langerhans was intact but the model or untreated group underwent severe degeneration in the pancreatic tissue showing focal necrosis in the area. Metformin and both 500 mg/kg and 800 mg/kg doses of extract restored the boundary of islets of Langerhans as well as the morphology and number of beta cells that are involved in insulin secretion.

## 4. Discussion

T2DM is a multifactorial disease with complex pathogenesis and is mainly classified into impaired insulin secretion and action [2]. Despite having many medications approved for T2DM therapy, the prevalence is still rising and current approved drugs are facing challenges of serious side effects [14]. Many medicinal plants, having a plethora of phytobiologics, are being evaluated for their antidiabetic effect in search for suitable medication against T2DM. *Thymus serpyllum*, a well-known aromatic plant of the Himalayan region, is often used in pharmaceutical industries because of its phenolic and non-phenolic constituents. Previous studies on the essential oil of *Thymus serpyllum* confirmed the presence of flavonoids, terpenoids, and phenolic acid [33,34,35]. Moreover, non-phenolic compounds such as linalool and *p*-cymene were also found in essential oil of Thyme. Recently, Galovičová et al. analyzed the composition of essential oil of *Thymus serpyllum* and reported the presence of thymol, 18.8%; carvacrol, 17.4%; o-cymene, 15.4%; and geraniol, 10.7% as major chemical constituents [36]. Similarly, Goyal et al. studied the volatile constituents of *Thymus serpyllum* harvested from different regions of west Himalayas, using gas chromatography-mass spectrometry (GCMS) analysis. Thymol was the major compound present in all cultivations. However, camphor, alpha-thujene, alpha-terpineol, *p*-cymene, beta-bisabolene, (*E*)-caryophyllene, alpha-pinene, and carvacrol were also identified in significant amounts [37].

*Thymus serpyllum* has been known for its antimicrobial [38], anti-tumor [39], and anti-arthritic effects [24]. For diabetes, preventive effects of *Thymus serpyllum* were evaluated [40] but the plant and its constituents have not been explored yet for their therapeutic efficacy and no molecular mechanism for its mode of action has been defined. Herein, we evaluated the therapeutic efficacy of *Thymus serpyllum* to attenuate hyperglycemia in streptozotocin-induced type 2 diabetic mice. Results indicated that treatment with aqueous extract improved the glucose homeostasis by altering the expression of *IRS1*, *GLUT2* and *AMPK* genes due to attributes of its chemical constituents. Plants belonging to genus Thymus exhibit anti-inflammation and antioxidative properties [25]. We studied the antioxidant activity of aqueous extract of *Thymus serpyllum* through DPPH assay which revealed the significant free radical scavenging potential. Ruiz-Malagón et al. studied the antioxidative effects of *Thymus serpyllum* in high-fat diet obese mice through DPPH assay [41]. The extract showed the neutralization of free radicals maximally 54.3% at the concentration of 100 mg/mL. In our study, *Thymus serpyllum* aqueous extract displayed the radical scavenging activity maximally 75.5% at 100 µg/mL. In our study, thyme extract also showed the alpha amylase inhibition potential which represents that it can cause delay in digestion of carbohydrates through which post-prandial hyperglycemia can be managed. These attributes are all due to the bioactive compounds present in the extract. The extract successfully alleviates the hyperglycemia in STZ-induced diabetic mice when administered orally and pose significant effect on body weight.

Insulin insensitivity and insulin insufficiency are both leading causes of insulin resistance in the body [42]. Insulin defect and less glucose tolerance can lead to chronic hyperglycemia which will disrupt many signaling cascades cause the body tissues to utilize glucose in blood. We found that aqueous extract of *Thymus serpyllum* improved the glucose and insulin tolerance in the body of mice. Recently, Ruiz-Malagón et al. analyzed the effects of *Thymus serpyllum* extract on obesity-associated metabolic alterations [41]. They evaluated the impact of thyme extract on glucose tolerance in obese mice. Thyme extract treatment reduced the glucose levels from 30 min onward and hence, the area under the curve (AUC) was significantly reduced in the treatment group. Similarly, in our study, *Thymus serpyllum* extract reduced the fasting blood glucose levels in the treatment groups from 30 min onward and AUC was significantly reduced in comparison with the untreated diabetic group. Hence, the results of *Thymus Serpyllum* extract in improving glucose tolerance in vivo in our study were supported by the study of Ruiz-Malagón et al. [41].

Furthermore, the present study also presented that aqueous extract improves the insulin action through regulating the expression of *IRS1* and *AMPK* genes in liver tissue. *AMPK* and *IRS1* signaling are very crucial in regulating glucose homeostasis. *AMPK* reduced hyperglycemia by controlling hepatic glucose production. *AMPK* was also shown to activate the insulin signaling by activating the glucose transporter 4 (*GLUT4*) and *IRS1* that control the regulation of body glycemic state [9,43]. *IRS1* is an insulin receptor substrate whose activation initiates signaling cascade where different proteins interact and activate to open channels for glucose transportation in tissues. The administration of thyme extract resulted in a significantly increased expression of *IRS1* in the liver, thus confirming the enhancement in insulin signaling, as evidenced by the improvement of blood glucose levels in treated-diabetic mice. Similarly, mice groups treated with thyme extract showed an amelioration in the expression of *AMPK* via its upregulation in treated groups, thus revealing that the increased expression of *AMPK* observed in treated mice groups was associated with normal glucose metabolism and controlled hepatic glucose production. Metformin-treated mice also revealed similar effects in regulating gene expression levels. Previous studies on evaluating the efficacy of rosmarinic acid, a chemical constituent of thyme extract, also showed improved insulin sensitivity in different metabolic organs, such as liver and skeletal muscle, via activation of *AMPK* [44]. Similarly, rosmarinic acid and carnosic acid (diterpene in thyme extract) are reported to improve insulin sensitivity in diabetic rabbits via upregulation of HO-1 and to induce anti-inflammatory and antidiabetic effects on mouse organoids by activation of *Nrf2* and inhibiting the *NF-κB* [45,46]. Moreover, thyme essential oil rich in monoterpenes is reported to upregulate the expression of *Hmox1* and *Nrf2*, which are known to play an important role in cellular protection against oxidative stress in diabetes and cardiovascular diseases [47]. Furthermore, Saravanan and Pari analyzed the antihyperglycemic effects of thymol in diabetic C57BL/6J mice fed with a high-fat diet. Daily intragastric application of thymol (40 mg/kg body weight) for 5 weeks caused a significant decline in plasma glucose, insulin resistance, HbA1c, and leptin [48]. Consequently, the phenolic compounds in thyme extract such as rosmarinic acid and thymol can contribute to improving glucose tolerance in T2DM.

Further, to have insight on the effect of aqueous extract on liver, kidney, and pancreas, we found that treatment with thyme extract restore and improved the symptoms of hepatocyte hypertrophy, focal necrosis, and renal hemorrhage as compared to the diabetic mice group which revealed these symptoms. Our results in the present study are also consistent with the results obtained from other plants of genus Thymus. A study on the aqueous extract of *Thymus satureioides* was conducted to investigate its antidiabetic potential in streptozotocin-induced diabetic rats [49]. Results showed that administration of aqueous extract from *Thymus satureioides* for 28 days reduced hyperglycemia in diabetic rats and improved body weight and glucose tolerance. Another study performed on the methanolic extract of *Thymus vulgaris* demonstrated antidiabetic activity by inhibition of α-amylase and α-glucosidase [50]. In our study, the observed antidiabetic effect may be attributed to chemical constituents of *Thymus serpyllum* such as polyphenols but cannot be directly related until further explored with specific compounds and other markers. The aqueous extract of *Thymus serpyllum* should be characterized further for identification of bioactive compounds present in the extract and further explored for its therapeutic potential in type 2 diabetes associated complications through alternative pathways.

## 5. Conclusions

The present study reveals the antidiabetic potential of phytochemicals derived from *Thymus serpyllum* in streptozotocin-induced type 2 diabetic mice. *Thymus serpyllum* extract showed significant antioxidant activity to reduce the burden of free radicals in vitro. Moreover, the extract showed the potential to inhibit alpha amylase to manage post-prandial hyperglycemia. Furthermore, the extract at a dose of 500 mg/kg and 800 mg/kg showed significant reduction in blood glucose levels and improved the glucose and insulin tolerance in mice models when administered in diet. Our findings showed that *Thymus serpyllum* extract has a significant impact on the regulation of the expression of *AMPK* and *IRS1* genes, which aid in glucose uptake and insulin sensitization. Thyme extract also showed a restorative impact on the cellular morphology of the liver, kidney, and pancreas. Hence, we suggested that *Thymus serpyllum* could be used as a promising feed additive to improve the signs and symptoms of type 2 diabetes. However, further studies are needed to elucidate the molecular mechanism of action by targeting other possible pathways and validate our findings.

## Figures and Tables

**Table 1:** Phytochemical screening of aqueous extract of *Thymus serpyllum*.

Phytochemical	Aqueous Extract
Alkaloids	**++**
Phenols	**+++**
Flavonoids	**++**
Anthraquinones	**++**
Anthocyanins	**-**
Phlobatannins	**+**
Coumarins	**++**
Terpenoids	**+**
Sterols	**++**
Steroids	**+++**
Saponins	**-**
Glycosides	**+**
Tannins	**+++**
Amino acids	**+++**
Carbohydrates	**++**

**-** Not present, **+** weakly present, **++** moderately present, **+++** strongly present.

## Data Availability

Data are available on request to the corresponding author.

## Supplementary Materials

The following supporting information can be downloaded at: https://www.mdpi.com/article/10.3390/nu14173561/s1, Table S1: Nucleotide sequence and properties of primers used for qRT-PCR analysis.

## Abbreviations

FBG	Fasting blood glucose
HFD	High-fat diet
MET	Metformin
TS-AQ	Aqueous extract of *Thymus serpyllum*
T2DM	Type 2 diabetes mellitus
AUC	Area under the curve

## References

[1] Lin Y., Sun Z. (2010). Current views on type 2 diabetes. J. Endocrinol..

[2] Chatterjee S., Khunti K., Davies M.J. (2017). Type 2 diabetes. Lancet.

[3] Zheng Y., Ley S.H., Hu F.B. (2018). Global aetiology and epidemiology of type 2 diabetes mellitus and its complications. Nat. Rev. Endocrinol..

[4] Shulman G.I. (2000). Cellular mechanisms of insulin resistance. J. Clin. Investig..

[5] Carling D. (2004). The AMP-activated protein kinase cascade—A unifying system for energy control. Trends Biochem. Sci..

[6] Foretz M., Even P.C., Viollet B. (2018). AMPK activation reduces hepatic lipid content by increasing fat oxidation in vivo. Int. J. Mol. Sci..

[7] Lochhead P.A., Salt I.P., Walker K.S., Hardie D.G., Sutherland C. (2000). 5-aminoimidazole-4-carboxamide riboside mimics the effects of insulin on the expression of the 2 key gluconeogenic genes PEPCK and glucose-6-phosphatase. Diabetes.

[8] Cool B., Zinker B., Chiou W., Kifle L., Cao N., Perham M., Dickinson R., Adler A., Gagne G., Iyengar R. (2006). Identification and characterization of a small molecule AMPK activator that treats key components of type 2 diabetes and the metabolic syndrome. Cell Metab..

[9] Chopra I., Li H., Wang H., Webster K. (2012). Phosphorylation of the insulin receptor by AMP-activated protein kinase (AMPK) promotes ligand-independent activation of the insulin signalling pathway in rodent muscle. Diabetologia.

[10] Carvalho E., Jansson P.-A., Axelsen M., Eriksson J.W., Huang X., Groop L., Rondinone C., Sjostrom L., Smith U.P. (1999). Low Cellular IRS 1 Gene and Protein Expression Predict Insulin Resistance and Type 2 Diabetes. Diabetes.

[11] Hirosumi J., Tuncman G., Chang L., Görgün C.Z., Uysal K.T., Maeda K., Karin M., Hotamisligil G.S. (2002). A central role for JNK in obesity and insulin resistance. Nature.

[12] Thorens B. (2015). GLUT2, glucose sensing and glucose homeostasis. Diabetologia.

[13] Hoffmann I.S., Roa M., Torrico F., Cubeddu L.X. (2003). Ondansetron and metformin-induced gastrointestinal side effects. Am. J. Ther..

[14] Marín-Peñalver J.J., Martín-Timón I., Sevillano-Collantes C., del Cañizo-Gómez F.J. (2016). Update on the treatment of type 2 diabetes mellitus. World J. Diabetes.

[15] Sanchez-Rangel E., Inzucchi S.E. (2017). Metformin: Clinical use in type 2 diabetes. Diabetologia.

[16] Rena G., Hardie D.G., Pearson E.R. (2017). The mechanisms of action of metformin. Diabetologia.

[17] LaMoia T.E., Shulman G.I. (2021). Cellular and Molecular Mechanisms of Metformin Action. Endocr. Rev..

[18] Musi N., Hirshman M.F., Nygren J., Svanfeldt M., Bavenholm P., Rooyackers O., Zhou G., Williamson J.M., Ljunqvist O., Efendic S. (2002). Metformin increases AMP-activated protein kinase activity in skeletal muscle of subjects with type 2 diabetes. Diabetes.

[19] Wu H., Esteve E., Tremaroli V., Khan M.T., Caesar R., Mannerås-Holm L., Ståhlman M., Olsson L.M., Serino M., Planas-Fèlix M. (2017). Metformin alters the gut microbiome of individuals with treatment-naive type 2 diabetes, contributing to the therapeutic effects of the drug. Nat. Med..

[20] Bouchoucha M., Uzzan B., Cohen R. (2011). Metformin and digestive disorders. Diabetes Metab..

[21] Jabeen N., Ajaib M., Siddiqui M.F. (2015). A survey of ethnobotanically important plants of district Ghizer, Gilgit-Baltistan. FUUAST J. Biol..

[22] Salehi B., Mishra A.P., Shukla I., Sharifi-Rad M., Contreras M.d.M., Segura-Carretero A., Fathi H., Nasrabadi N.N., Kobarfard F., Sharifi-Rad J. (2018). Thymol, thyme, and other plant sources: Health and potential uses. Phytother. Res..

[23] Jarić S., Mitrović M., Pavlović P. (2015). Review of ethnobotanical, phytochemical, and pharmacological study of *Thymus serpyllum* L.. Evid. Based Complement. Altern. Med..

[24] Jannat A., John P., Bhatti A., Hayat M.Q. (2019). Tomorou attenuates progression of rheumatoid arthritis through alteration in ULK-1 independent autophagy pathway in collagen induced arthritis mice model. Cell Death Discov..

[25] Algieri F., Rodriguez-Nogales A., Garrido-Mesa N., Zorrilla P., Burkard N., Pischel I., Sievers H., Benedek B., Feistel B., Walbroel B. (2014). Intestinal anti-inflammatory activity of the Serpylli herba extract in experimental models of rodent colitis. J. Crohn’s Colitis.

[26] Fachini-Queiroz F.C., Kummer R., Estevao-Silva C.F., Carvalho M.D.d.B., Cunha J.M., Grespan R., Bersani-Amado C.A., Cuman R.K.N. (2012). Effects of thymol and carvacrol, constituents of Thymus vulgaris L. essential oil, on the inflammatory response. Evid. Based Complement. Altern. Med..

[27] Tsuji T., Mori T., Taniguchi M., Shimizu K., Kobayashi T. (1985). Solvent extraction of plant pigments from leaf protein concentrate. J. Chem. Eng. Jpn..

[28] Kaur G.J., Arora D.S. (2009). Antibacterial and phytochemical screening of Anethum graveolens, Foeniculum vulgare and Trachyspermum ammi. BMC Complement. Altern. Med..

[29] Sanganna B., Chitme H.R., Vrunda K., Jamadar M.J. (2016). Antiproliferative and antioxidant activity of leaves extracts of Moringa oleifera. Int. J. Curr. Pharm. Res..

[30] McCue P.P., Shetty K. (2004). Inhibitory effects of rosmarinic acid extracts on porcine pancreatic amylase in vitro. Asia Pac. J. Clin. Nutr..

[31] Noor A., Zahid S. (2017). Alterations in adult hippocampal neurogenesis, aberrant protein s-nitrosylation, and associated spatial memory loss in streptozotocin-induced diabetes mellitus type 2 mice. Iran. J. Basic Med. Sci..

[32] Shin J.-W., Seol I.-C., Son C.-G. (2010). Interpretation of animal dose and human equivalent dose for drug development. J. Korean Med..

[33] Verma R.S., Rahman L.U., Chanotiya C.S., Verma R.K., Singh A., Yadav A., Chauhan A., Yadav A.K., Singh A.K. (2009). Essential oil composition of *Thymus serpyllum* cultivated in the Kumaon region of western Himalaya, India. Nat. Prod. Commun..

[34] Verma R., Verma R., Chauhan A., Yadav A. (2011). Seasonal variation in essential oil content and composition of Thyme, *Thymus serpyllum* L. cultivated in Uttarakhand Hills. Indian J. Pharm. Sci..

[35] Nikolić M., Glamočlija J., Ferreira I.C., Calhelha R.C., Fernandes Â., Marković T., Marković D., Giweli A., Soković M. (2014). Chemical composition, antimicrobial, antioxidant and antitumor activity of *Thymus serpyllum* L., Thymus algeriensis Boiss. and Reut and Thymus vulgaris L. essential oils. Ind. Crops Prod..

[36] Galovičová L., Borotová P., Valková V., Vukovic N.L., Vukic M., Terentjeva M., Štefániková J., Ďúranová H., Kowalczewski P.Ł., Kačániová M. (2021). *Thymus serpyllum* Essential Oil and Its Biological Activity as a Modern Food Preserver. Plants.

[37] Goyal S., Pathak R., Pandey H.K., Kumari A., Tewari G., Bhandari N.S., Bala M. (2020). Comparative study of the volatile constituents of *Thymus serpyllum* L. grown at different altitudes of Western Himalayas. SN Appl. Sci..

[38] Abramovic H., Abram V., Cuk A., Ceh B., SMOLE-MOZINA S., Vidmar M., Pavlovic M., ULRIH N.P. (2018). Antioxidative and antibacterial properties of organically grown thyme (*Thymus* sp.) and basil (*Ocimum basilicum* L.). Turk. J. Agric. For..

[39] Aralbaeva A.N., Mamataeva A.T., Zhaparkulova N.I., Utegalieva R.S., Khanin M., Danilenko M., Murzakhmetova M.K. (2017). A composition of medicinal plants with an enhanced ability to suppress microsomal lipid peroxidation and a protective activity against carbon tetrachloride-induced hepatotoxicity. Biomed. Pharm..

[40] Mushtaq M.N., Bashir S., Ullah I., Karim S., Rashid M., Hayat Malik M.N. (2016). Comparative hypoglycemic activity of different fractions of *Thymus serpyllum* L. in alloxan induced diabetic rabbits. Pak. J. Pharm. Sci..

[41] Ruiz-Malagón A.J., Rodríguez-Sojo M.J., Hidalgo-García L., Molina-Tijeras J.A., García F., Pischel I., Romero M., Duarte J., Diez-Echave P., Rodríguez-Cabezas M.E. (2022). The Antioxidant Activity of *Thymus serpyllum* Extract Protects against the Inflammatory State and Modulates Gut Dysbiosis in Diet-Induced Obesity in Mice. Antioxidants.

[42] Lucidi P., Rossetti P., Porcellati F., Pampanelli S., Candeloro P., Andreoli A.M., Perriello G., Bolli G.B., Fanelli C.G. (2010). Mechanisms of insulin resistance after insulin-induced hypoglycemia in humans: The role of lipolysis. Diabetes.

[43] Habegger K.M., Hoffman N.J., Ridenour C.M., Brozinick J.T., Elmendorf J.S. (2012). AMPK enhances insulin-stimulated GLUT4 regulation via lowering membrane cholesterol. Endocrinology.

[44] Naimi M., Vlavcheski F., Shamshoum H., Tsiani E. (2017). Rosemary extract as a potential anti-hyperglycemic agent: Current evidence and future perspectives. Nutrients.

[45] Elbadawi M., Ammar R.M., Aziz-Kalbhenn H., Rabini S., Klauck S.M., Dawood M., Saeed M.E., Kampf C.J., Efferth T. (2021). Anti-inflammatory and tight junction protective activity of the herbal preparation STW 5-II on mouse intestinal organoids. Phytomedicine.

[46] Xie Z., Zhong L., Wu Y., Wan X., Yang H., Xu X., Li P. (2018). Carnosic acid improves diabetic nephropathy by activating Nrf2/ARE and inhibition of NF-κB pathway. Phytomedicine.

[47] He T., Li X., Wang X., Xu X., Yan X., Li X., Sun S., Dong Y., Ren X., Liu X. (2020). Chemical composition and anti-oxidant potential on essential oils of Thymus quinquecostatus Celak. from Loess Plateau in China, regulating Nrf2/Keap1 signaling pathway in zebrafish. Sci. Rep..

[48] Saravanan S., Pari L. (2015). Role of thymol on hyperglycemia and hyperlipidemia in high fat diet-induced type 2 diabetic C57BL/6J mice. Eur. J. Pharm..

[49] Kabbaoui M., Chda A., Mejrhit N., Azdad O., Farah A., Aarab L., Bencheikh R., Tazi A. (2016). Antidiabetic effect of Thymus satureioides aqueous extract in streptozotocin-induced diabetic rats. Int. J. Pharm. Pharm. Sci..

[50] Aljarah A.K., Hameed I.H. (2018). In Vitro anti-diabetic properties of Methanolic extract of Thymus vulgaris using α-glucosidase and α-amylase inhibition assay and determination of its bioactive chemical compounds. Indian J. Public Health Res. Dev..

