# Supplementary material for: Thymus serpyllum Exhibits Anti-Diabetic Potential in Streptozotocin-Induced Diabetes Mellitus Type 2 Mice: A Combined Biochemical and In Vivo Study
Source: Nutrients. 2022 Aug 29;14(17):3561. doi: 10.3390/nu14173561 (PMC9460602; doi:10.3390/nu14173561)
Supplement: Supplementary file 1 [file nutrients-14-03561-s001.zip › nutrients-1848252-supplementary.pdf]

Supplementary Material

Table S1. Nucleotide sequence and properties of primers used for qRT-PCR analysis.

Gene	Sequence 5′ to 3′	Reference
AMPK-F	GTCGACGTAGCTCCAAGACC	[21]
AMPK-R	ATCGTTTTCCAGTCCCTGTG	
IRS1-F	ACATCACAGCAGAATGAAGACC	
IRS1-R	CCGGTGTCACAGTGCTTTCT	[41]
GLUT2-F	TCAGAAGACAAGATCACCGGA	
GLUT2-R	GCTGGTGTGACTGTAAGTGGG	
GAPDH-F	ACCCAGAAGACTGTGGATGG	[22]
GAPDH-R	CACATTGGGGGTAGGAACAC	
